# Carotid Bifurcation With Tandem Stenosis—A Patient-Specific Case Study Combined *in vivo* Imaging, *in vitro* Histology and *in silico* Simulation

**DOI:** 10.3389/fbioe.2019.00349

**Published:** 2019-11-20

**Authors:** Jiaqiu Wang, Phani Kumari Paritala, Jessica Benitez Mendieta, Yuantong Gu, Owen Christopher Raffel, Tim McGahan, Thomas Lloyd, Zhiyong Li

**Affiliations:** ^1^School of Chemistry, Physics and Mechanical Engineering, Queensland University of Technology, Brisbane, QLD, Australia; ^2^Department of Cardiology, The Prince Charles Hospital, Brisbane, QLD, Australia; ^3^School of Medicine, University of Queensland, Brisbane, QLD, Australia; ^4^Department of Vascular Surgery, Princess Alexandra Hospital, Brisbane, QLD, Australia; ^5^Department of Radiology, Princess Alexandra Hospital, Brisbane, QLD, Australia; ^6^School of Biological Science & Medical Engineering, Southeast University, Nanjing, China

**Keywords:** atherosclerosis, carotid bifurcation, computational fluid dynamics (CFD), histology, tandem stenosis

## Abstract

A patient-specific carotid bifurcation with tandem stenosis found at both internal carotid artery (ICA) and common carotid artery (CCA) was studied. The *in vivo* pre-carotid endarterectomy (pre-CEA) multi-spectral magnetic resonance imaging (MRI) were performed and *in vitro* post-CEA carotid plaque tissue sample was collected. MR imaging data and tissue sample staining histology were used to recognize the plaque components. Further, the computational fluid dynamics (CFD) were performed on four MR-based reconstructed 3D carotid bifurcation models (the patient-specific geometry with tandem stenosis and three presumptive geometries by removing the stenosis part). The flow and shear stress behavior affected by the tandem stenosis was analyzed. From the results of MR segmentation and histology analysis, plaque lipid pool and calcification were found at both ICA and CCA. From the result of CFD simulation, the flow shear stress behavior suggested the tandem stenosis as a more “dangerous” situation than a single-stenosis artery. Besides, the CFD results deduced that the stenosis at the CCA location formed initially and led to the subsequent formation of stenosis at ICA. This study suggests that when planning CEA, CFD simulation on the presumptive models could help clinicians to estimate the blood flow behavior after surgery. Particular attention should be paid to the case of tandem stenosis, as the local hemodynamic environment is more complex and treatment of one stenosis may lead to a variation in the hemodynamic loading on the second plaque, which may result in either a higher risk of plaque rupture or restenosis.

## Introduction

Stroke is one of the leading causes of disability and death worldwide (Benjamin et al., 2019). Carotid stenosis is known as one of the main risk factors to the ischemic stroke, which contributes over three quarters of the total stroke cases (Markus, 2016). To prevent the ischemic stroke, carotid endarterectomy (CEA) and carotid-artery stenting (CAS) are options for treating carotid artery stenosis (Brott et al., 2010), and CEA is considered as the gold standard treatment of carotid stenosis (European Carotid Surgery Trialists' Collaborative Group, 1998; Ferguson et al., 1999; Thomas et al., 2018).

In some cases, it can be found more than one location with stenosis in the patient's carotid artery, which is called tandem stenosis. Most reports of tandem stenosis occurred at internal carotid artery (ICA) and middle cerebral artery (MCA) (El-Mitwalli et al., 2002; Kim et al., 2005; Rubiera et al., 2006), in which the highest occurrence was 31%. In a case review report, the tandem stenosis occurred at ICA and common carotid artery (CCA), same situation as in this study, the frequency was only 2.1% (14 out of 672 patients). Despite the relatively low frequency of tandem stenosis, the authors suggested that tandem stenosis had important implications for long-term medical management even after endarterectomy (Lee et al., 2008).

To study the tandem stenosis phenomenon, several studies have been published by using numerical methods. In our previous study, Li et al. (2010) simulated an idealized blood vessel model with tandem stenosis by using finite volume method (FVM) and suggested that between two stenosis there was a low wall shear stress (WSS) area where might merge the two stenosis into a large lesion. Park et al. (2012) used an idealized tube model with tandem stenosis to validate the use of fractional flow reserve gradient (ΔFFR) in coronary treatment. Another report of using idealized tandem stenosis model was from Rabby et al. (2014), they compared the risk of thrombogenesis at the downstream of stenosis by using Newtonian and non-Newtonian flow properties. In these studies, the models used were all idealized tube shape without bifurcation. There is a lack of report of studying tandem stenosis using patient-specific geometry with bifurcation. Besides, to our knowledge, there is not a report related to this topic combined the *in vivo* imaging, *in vitro* histology, and *in silico* simulation together yet.

In this study, we recruited a patient with a tandem carotid stenosis at both ICA and CCA. The collected data included multi-sequence pre-CEA magnetic resonance (MR) imaging and post-CEA carotid plaque tissue sample. We performed MR imaging processing and reconstruction, tissue sample staining for plaque recognition purpose. We also reconstructed 3D patient-specific carotid bifurcation from MR imaging. Using the reconstructed carotid models, the pulsatile flow computational fluid dynamics (CFD) simulation was conducted to investigate how the tandem stenosis developed and affected each other. The purpose of this study was to investigate the impact of the CEA on the hemodynamics in patients with a tandem stenosis.

## Methods

### Data Acquisition

The data and sample used in this study were from a patient (male, 76 years old) with a tandem stenosis at his common carotid artery (CCA) and internal carotid artery (ICA). The patient had the CEA in the Prince Alexandra Hospital (PAH, Brisbane, QLD 4000, Australia). This study was approved by the Metro South Human Research Ethics Committee (HREC/17/QPAH/181) and patient content form was obtained.

Multi-sequence pre-CEA three-dimensional (3D) *in vivo* magnetic resonance imaging (MRI) data for morphologic segmentation and 3D geometry reconstruction; 2D cine Phase Contrast (PC) for patient-specific boundary conditions extraction.

**Figure 1:**
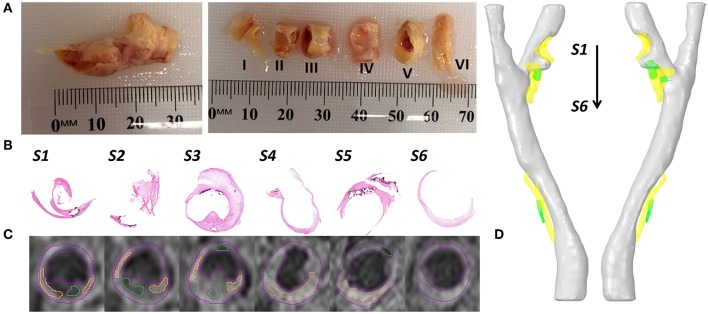
Examples of collected data and data processing. **(A)** The collected carotid plaque tissue sample (left) and later the sample was cut into six segments with 3–4 mm thick. **(B)** The Hematoxylin & Eosin (H & E) staining of 6 slices. **(C)** At the same position of staining slices, the T1-weighted MR imaging slices with segmented contours (lumen and vessel in red color, lipid in yellow color, and calcification in green color). **(D)** The reconstructed 3D patient-specific carotid bifurcation, including the geometries of lumen (gray), lipid (yellow) and calcification (green).

After CEA, the carotid plaque tissue sample was collected (Figure 1A). The sample was firstly placed in phosphate buffered saline (PBS) and transported to Queensland University of Technology's (QUT) Institute of Health and Biomedical Innovation (IHBI) for histology processing.

### Histology Analysis

The tissue sample was cut into 3–4 mm thick segments (Figure 1A) and placed in Optimal Cutting Temperature (OCT) compound (Tissue-Tek OCT, TED Pella Inc, CA, USA). Following that, the tissue samples were frozen using liquid Nitrogen. For staining, 8 μm thick slices were sectioned using a cryostat (Leica CM1850). Hematoxylin & Eosin (H & E, Figure 1B), Oil Red O and Masson's Trichrome Staining were used to define the fibrous tissue, lipids, calcification, and hemorrhage. Lipids were stained red in Oil Red O stain, and hemorrhage was identified both in H & E and Masson's Trichrome staining in dark pink color. Calcium deposits were lost in the sample preparation process but could be easily defined from both H & E and Oil Red O staining as they clearly show the boundary of calcification in dark purple color.

### Geometric Model

**Figure 2:**
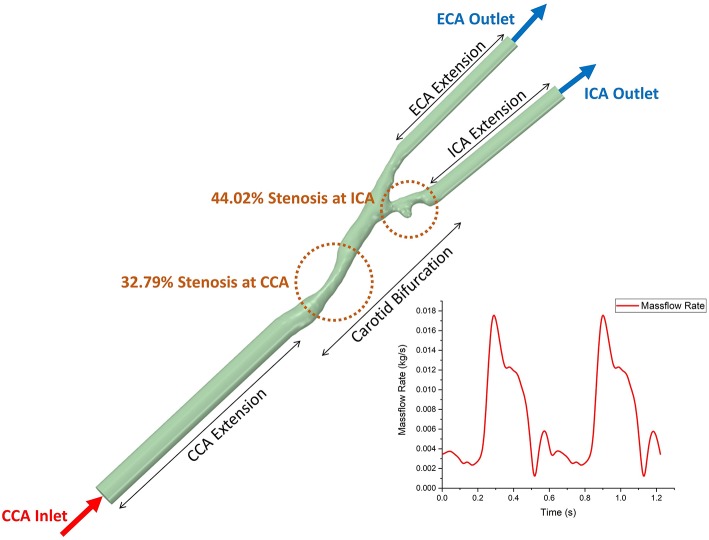
The geometric model of 3D patient-specific carotid bifurcation with tandem stenosis for CFD simulation and the boundary conditions. The inlet and two outlets were extended to get fully developed flow profile. The time-dependent massflow rate boundary condition (extracted from phase-contrast MRI) was given to the CCA inlet, and the constant pressure profile was given to both ICA and ECA outlets.

The geometric model in this study was reconstructed from magnetic resonance imaging (MRI) data. The image processing software Amira (version 6.0, Thermo Fisher Scientific) was used for imaging processing, contour segmentation, and 3D reconstruction. In detail, the lumen geometric model, which was used for CFD simulation (Figure 2) was segmented and reconstructed from time of flight (ToF) sequence MR images. The plaque components (lipids and calcification) were segmented and reconstructed from a T1-weighted sequence of MR images (Figure 1C).

The reconstructed geometry of the patient-specific carotid bifurcation is shown in Figure 1D. It was found that two stenosis existed at the bifurcation and the ICA branch location and divided by a small protuberance. As the two stenosis at the ICA branch were very close to each other, thus they were treated as one single stenotic position. The degree of stenosis was calculated by using the method from North American Symptomatic Carotid Endarterectomy Trial (NASCET) (1991). For visualization, plaque components were segmented and reconstructed at the corresponding stenosis positions (lipid in yellow color and calcification in green color).

**Figure 3:**
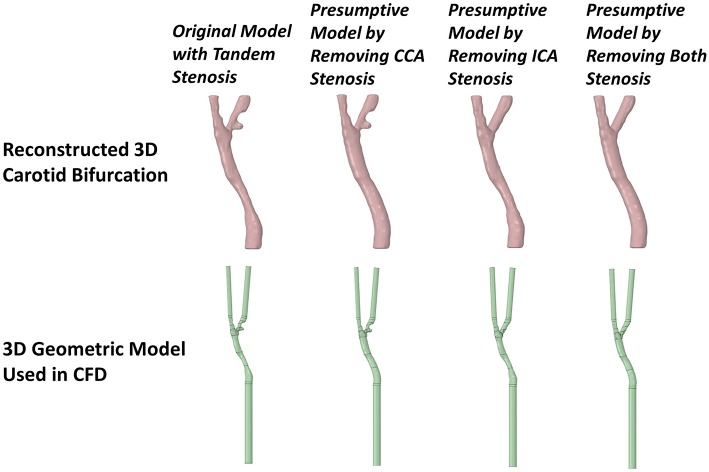
The 3D reconstructed patient-specific carotid bifurcation models and the geometric models used in CFD. From the left to right are, the original model with tandem stenosis, the presumptive model by removing the stenosis at CCA, ICT and both locations, respectively. The inlet and outlets were extended in the CFD geometric models for the purpose of flow fully-developing.

After the reconstructed 3D patient-specific carotid bifurcation lumen model with tandem stenosis, three presumptive models were created by removing the CCA stenosis, ICA stenosis, and both stenosis, respectively (Figure 3). The presumptive model with no stenosis (two stenosis removed) was assumed as a healthy carotid bifurcation. These presumptive models were used for fluid dynamics simulation. The results were used to compare with the patient-specific tandem stenosis model to discuss the plaque vulnerability and the tandem stenosis formation. Moreover, the presumptive models could be used to mimics the post-surgery blood flow behavior when planning CEA.

### Computational Dynamics Fluid (CFD) Model

In the CFD simulation, blood flow was assumed as incompressible and Newtonian. The value of viscosity was 0.00345 Pa·s, and the value of density was 1,050 kg/m^3^ (Liu and Tang, 2011). Standard k-omega turbulence model was applied to the flow. The inlet and outlets of the bifurcation were extended, patient-specific massflow rate (acquired from PC MR data) profile was prescribed at the extended inlet and at both extended outlets were set as pressure-out (Figure 2). Non-slip boundary condition was applied at the lumen boundary. ANSYS Fluent (version 19.0, ANSYS Inc.) was used to solve the k-omega two equation turbulence model. The simulation time step size was set to 0.005 s, running 245 steps (1.22 s, equals to 2 cardiac cycles), the result data from the second cardiac cycle were used for post-process and data analysis.

### Analysis of CFD Results

The post-processing software ANSYS CFD-post (version 19.0, ANSYS Inc.) and the data analysis software Origin (version 2018, OriginLab Corp.) were used for results analysis and visualization. To understand the flow distribution at the bifurcation, the flowrates from ICA and ECA were compared. Wall shear stress (WSS) was used as an index to assess the hemodynamic behavior. A low value of WSS indicated an atherosclerosis-prone area, while a high value of WSS might promote the development of high-risk plaques (Dolan et al., 2013; Eshtehardi et al., 2017). Besides, several WSS-based derived index were used in this study. Time-averaged WSS (TaWSS) is defined as,

$$\begin{array}{l} \text{TaWSS}=\;\frac{1}{T}\int_{0}^{T}|\bar{\tau_{w}}\;|dt, \end{array}$$

where $\left|\bar{\tau_{w}}\right|$ is the instantaneous WSS magnitude and T is the cardiac cycle period.

Oscillatory shear index (OSI) describes the difference between WSS acting in directions and the direction of temporal mean WSS vector (Ku et al., 1985). OSI is defined as,

$$\begin{array}{l} \text{OSI}=\;\frac{1}{2}\left(1-\frac{\left|\int_{0}^{T}\bar{\tau_{w}}dt\right|}{\int_{0}^{T}|\bar{\tau_{w}}|dt}\;\right). \end{array}$$

Relative residence time (RRT) is marked by low WSS magnitude and high oscillatory WSS (Himburg et al., 2004), it is defined as

$$\begin{array}{l} \text{RRT}=\;\frac{1}{\left(1-2\cdot OSI\right)\cdot \;TaWSS}. \end{array}$$

The low value of TaWSS and high value of OSI and RRT are used as indicators to find out the atherogenesis region (Soulis et al., 2011).

## Results

### Plaque Visualization

**Figure 4:**
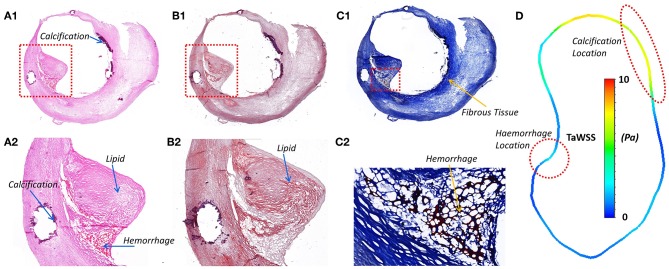
The histology staining at the ICA stenosis location, the lipid, calcification, and hemorrhage could be viewed in different staining. **(A1)** H & E staining; **(A2)** Calcium in dark purple and hemorrhage in dark pink; **(B1)** Oil Red O; **(B2)** Lipids stained in red; **(C1)** Masson's Trichrome; **(C2)** Fibrous tissue in blue and hemorrhage in dark pink. **(D)** The lumen TaWSS contour from CFD result at the position of histology slice. WSS was found to have an association with the location of hemorrhage and calcification.

Histological analysis was performed on the sections cut from the six segments of the carotid plaque tissue. The histological sections were matched to the MRI images based on the location of bifurcation and plaques (Figure 1). The lipids and calcifications were well-matched at the same location between MR and histological imaging. Moreover, histological staining provided further detail information about the variations between lipid, calcification, fibrous tissue and hemorrhage (Figure 4). Integrated with TaWSS plot of lumen contour, the hemorrhage location was associated with a moderate value of TaWSS, while the calcification area was found to have a high TaWSS present.

### Flow Distribution

**Figure 5:**
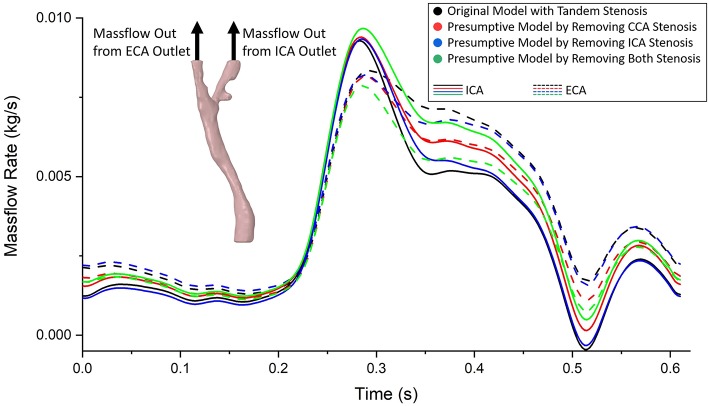
The massflow rate from ICA (solid line) and ECA (dashed line) outlets in the tandem stenosis model and the three presumptive models by removing stenosis at different locations. It was found the difference of flow distribution between each model enlarged at systole period, and reduced at diastole period.

The same boundary conditions were set as massflow inlet and pressure outlet for the original model and the three presumptive models. Therefore, when the geometry of fluid domain changed based on different stenosis conditions, the flow distributed to ICA and ECA varied between each model. The curves of massflow out from ICA and ECA in one cardiac cycle in the four models were plotted in Figure 5. Generally, all the massflow curves varied following the trend of the prescribed massflow inlet boundary condition. Taking the healthy carotid bifurcation model (in green color, the presumptive model by removing both stenosis) as the benchmark situation. At most time during the cardiac cycle, the massflow went through the ICA branch was higher than that through the ECA branch. It was because of the bigger size of blood vessel in the ICA branch. When a stenosis appeared at CCA location (curves in blue color), although the sizes of ICA and ECA branches remains the same, because the flow stream changed after the CCA stenosis, the blood flow distribution was affected by the CCA stenosis. Following, more flow had “chosen” the ECA branch instead of the ICA branch. In consequence, less flow in ICA consequently occurred the low WSS on the blood vessel wall, which might lead to the atherosclerosis at the ICA branch. If the healthy carotid bifurcation had a stenosis at ICA firstly (curves in red color), the difference of flow distribution between ICA and ECA branch became even, because the blood vessel size of ICA branch was no longer bigger than the ECA. Finally, when the stenosis occurred at both ICA and CCA locations (curves in black color), it was found a distinct difference on the ICA/ECA flow distribution compared to the healthy model, as more flow had “chosen” the ECA branch.

### Flow Behavior

**Figure 6:**
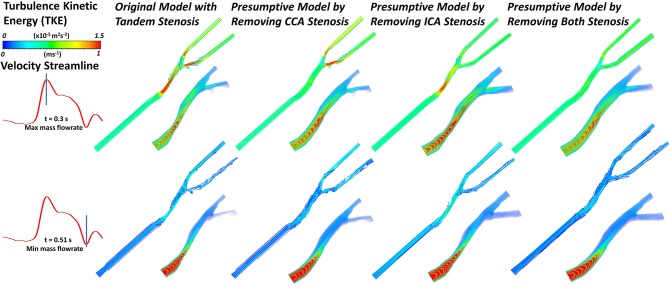
The velocity streamline and the volume rendering of turbulence kinetic energy (TKE) in the geometric models under the maximum and minimum mass flowrate (at timestep of ~0.3 and 0.51 s, respectively).

With different stenosis situation, the blood flow behavior also changed. The complexity of flow behavior affects the mechanical stress to the arterial wall, subsequently correlated to the stenosis formation and progression. In Figure 6, the blood flow streamline and turbulence kinetic energy (TKE) were plotted at the timestep with maximum and minimum mass flowrate. Turbulence kinetic energy (TKE) is an important variable related to the turbulence intensity. Basically, the TKE varied following the change of flow velocity. At apex points, there were always eddy flow at bifurcation location, also the TKE were relatively higher than surrounding locations. Besides, at the stenosis location, it proved the stenosis geometry was more likely to produce turbulence. The tandem stenosis showed more complex flow behavior than the other models. The velocity change rate also affected the flow behavior. For example, at the end-systole period, with the flowrate decreased, the proportion of eddy flow became more than that at the peak flowrate point.

### Wall Shear Stress (WSS)

**Figure 7:**
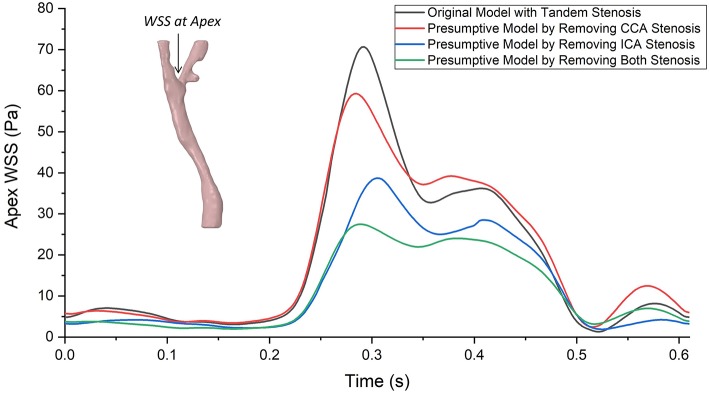
The highest values of wall shear stress (WSS) at bifurcation apex through one cardiac cycle in the tandem stenosis model and the three presumptive models by removing stenosis at different locations. Similar as the curves of massflow distribution, the difference of apex WSS between each model enlarged at systole period, and reduced at diastole period, The largest gap was found at around the ejection point (~0.3 s) between the tandem stenosis model (70.58 Pa) and the non-stenosis model (27.47 Pa).

Wall shear stress (WSS) is an important flow parameter which had a strong relationship to the atherosclerosis formation and plaque vulnerability. The highest value of WSS could always be found at the bifurcation apex. Figure 7 plotted the highest values of WSS at bifurcation apex through one cardiac cycle in the four carotid bifurcation models. The original patient-specific model with tandem stenosis (black) suffered the highest WSS at the apex. Nevertheless, the apex WSS in the presumptive model with only ICA stenosis model (red) was partially higher than the tandem stenosis model during the cardiac cycle. Compared with the presumptive model with only ICA stenosis model (red), the presumptive model with only CCA stenosis (blue) suffered a lower WSS at apex. The non-stenosis healthy model (green) had the lowest WSS value at the apex. The results show that the apex WSS increased following with the stenosis developing from non-stenosis to tandem stenosis. Also it is found that the stenosis at ICA had more impact on the apex WSS value than the stenosis at CCA. It was due to the stenosis at ICA branch was closer to the apex location, and it affected the blood flow at apex more directly than the stenosis at CCA.

**Figure 8:**
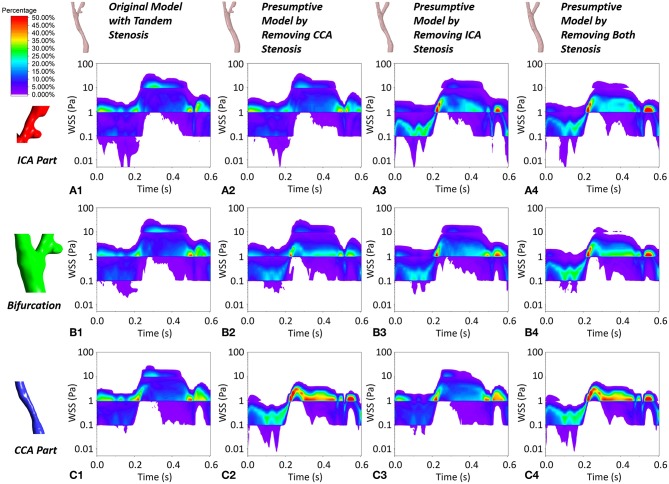
The contour maps of WSS concentration at local ICA part, local bifurcation part and local CCA part during one cardiac cycle in the four geometric models. **(A1–A4)** The WSS plot at local ICA part in the four geometric model. **(B1–B4)** The WSS plot at local bifurcation part in the four geometric model. **(C1–C4)** The WSS plot at local CCA part in the four geometric model.

Despite the WSS at the critical location (apex WSS), we selected three locations (ICA part, bifurcation part, and CCA part) and plotted the contour maps of WSS concentration during the cardiac cycle (Figure 8). The WSS concentration shifted following the trend of prescribed massflow rate boundary condition. Taking the non-stenosis healthy model as a benchmark, the WSS was mainly concentrated within a safe range (0.1–3 Pa) in all local parts. With the stenosis existing in the local part, the WSS concentration in the corresponding local part shifted up. When tandem stenosis formed in the model, the WSS concentrated in the high-value range, which was more than 10 Pa during the peak massflow rate reached in all the three local parts, which might lead to formation of a vulnerable plaque. It was also noticed that at the ICA local part (Figure 8A1), the tandem stenosis also had low WSS (<0.1 Pa) concentration more than other models, which indicated the potential atherosclerosis development.

**Figure 9:**
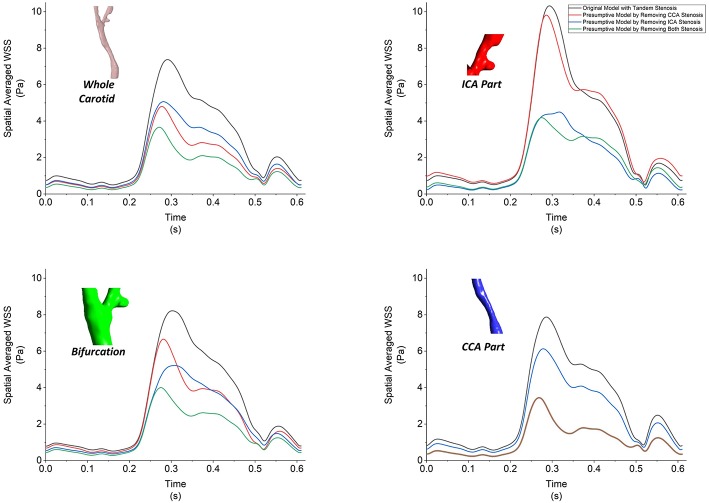
The spatial-averaged WSS at the surface of whole carotid model, local ICA part, local bifurcation part and local CCA part during one cardiac cycle for all the four models.

Besides the value of WSS, the time- and spatial-averaged WSS, oscillatory shear index (OSI) and relative residence time (RRT) are derived WSS parameters, which could help to understand the hemodynamic in stenotic carotid artery. Firstly we plotted the spatial-averaged WSS (SaWSS) at the whole carotid model, local ICA part, local bifurcation part and local CCA part during one cardiac cycle for all the four geometries (Figure 9). Generally, in the whole carotid geometries, the values of SaWSS during the cardiac cycle from high to low were: tandem stenosis model (black), single-CCA stenosis model (blue), single-ICA stenosis model (red), and non-stenosis healthy model (green). Then looking at the three local parts, at the local bifurcation part. Also the tandem stenosis model (black) suffered the high SaWSS, while the non-stenosis healthy model (green) had low values. The single stenosis model (single ICA-stenosis and single CCA-stenosis, red and blue) had a similar behavior in SaWSS during the cardiac cycle. At the local ICA and CCA parts, it was founded that the value of SaWSS depended on whether the stenosis existed in the location. For example, the high values of SaWSS in ICA part were found at the tandem stenosis model (black) and single-ICA stenosis model (red), and the high SaWSS in CCA part were at the tandem stenosis model (black) and single-CCA stenosis model (blue). It is worth mentioning that at the local CCA part, the curves of SaWSS from the models without CCA part (single-ICA stenosis model and non-stenosis model, red and green) were overlapped. It means the stenosis at upstream (ICA part) did not affect the WSS behavior at downstream (CCA part).

**Figure 10:**
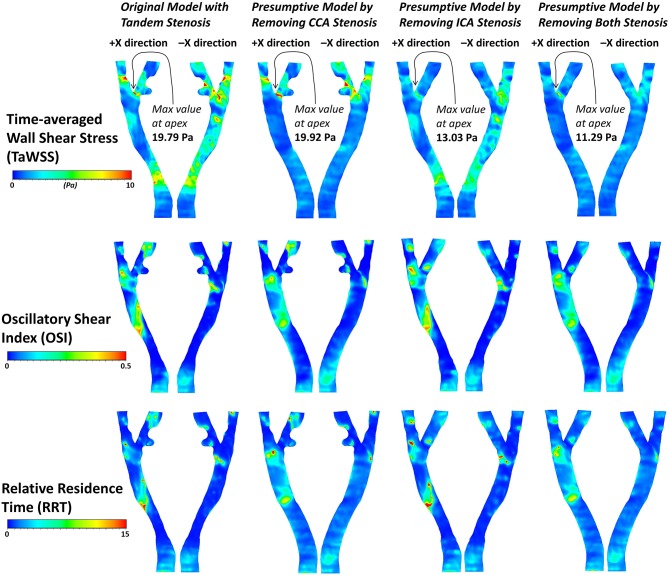
The contours of three derived WSS parameters, TaWSS, OSI and RRT in the four geometric models.

The contour of TaWSS, OSI, and RRT in the four models were plotted in Figure 10. The tandem stenosis model showed more area with high value of TaWSS (> 10 Pa) at the ICA and CCA stenosis location compared with the corresponding location in the single-stenosis models. It was found at the small protuberance on the ICA branch; there was a low TaWSS area might consequently cause atherosclerosis growing, which proved the previous result in Figure 8A1. For OSI and RRT, on the non-stenosis healthy model, the high OSI and RRT were found at CCA and bifurcation, which indicated at these locations the plaque might have developed. Then if considering that the stenosis firstly formed at the CCA part as the CCA single-stenosis model, the high OSI and RRT area appeared at the ICA branch location, where the stenosis formed at tandem stenosis model. Nevertheless, if the ICA stenosis was assumed to develop firstly as the ICA single-stenosis model, there was little difference at CCA part with the non-stenosis model. It was also previously deduced from the flow distribution result. Therefore, in this patient-specific carotid bifurcation, the stenosis at CCA location was more likely formed firstly and affected on the ICA stenosis formation.

## Discussion

### Development of Tandem Stenosis

The risk factor of low WSS and turbulent flow is associated with the atherosclerosis formation (Turan et al., 2010). From the CFD results comparing the two single-stenosis model, the CCA stenosis formation affected the downstream ICA area with low WSS, high OSI, and RRT, which might lead to secondary ICA stenosis and formatted the tandem stenosis. On the contrary, the formation of ICA stenosis had less influence on the upstream CCA stenosis. Another evidence is that the ICA location is one of the major stenosis distributions with about 25% among the major arteries (Turan et al., 2010). If the tandem stenosis was formed at ICA first and then affected at CCA location, the occurrence of tandem stenosis should not be as low as reported 2.1% (Lee et al., 2008).

This study indicates that the tandem stenosis may occur at the upstream location and then affected the downstream location. As the flow would be re-fully-developed after a length away from the stenosis, the location of the secondary stenosis formation would be close to the first stenosis. The flow behavior through the bifurcation might also assist on the formation of the tandem stenosis.

### Vulnerability of Tandem Stenosis

It was reported that a single-stenosis-repaired carotid artery was studied as a prospective observation. It was found that the hemodynamic environment and the WSS were found changed and might be related with the atherosclerosis formation and progression (Li et al., 2018). The tandem stenosis is more vulnerable than a single-stenosis, as the flow behavior is more complex after flow through the tandem stenosis, which might cause high or low WSS, cause the endothelial damage or the atherosclerosis formation. The tandem stenosis also changes the flow distribution at the bifurcation, which might lead to ischemic stroke. Moreover, as reported if the tandem stenosis is too close to each other, they might merge and format a large lesion (Li et al., 2010).

### Tandem Stenosis and CEA

The tandem stenosis is relatively uncommon and have not been reported to have much impact on the decision of CEA (Rouleau et al., 1999). However, we would like to suggest that when planning CEA, the tandem stenosis should be considered, because tandem stenosis makes the hemodynamic environment more complex in carotid bifurcation. Moreover, if tandem stenosis existed and only the ICA plaque was removed during CEA, the ICA stenosis is likely to be recurrent due to the CCA stenosis affection.

Besides, there is evidence of residual and recurrent stenosis after CEA because of the flow behavior changes (Bandyk et al., 1988). From our result and discussions, the upstream stenosis might affect downstream locations and cause second plaque after downstream stenosis had been removed during CEA. Therefore, when planning a CEA at carotid with tandem stenosis, the post-CEA flow behavior could be estimated by running CFD on the presumptive models. Clinicians might need to consider the flow behavior changes before and after surgery to avoid recurrent stenosis when planning the CEA.

### Highlights and Limitations

In this patient-specific case study, we evaluated the risk of carotid bifurcation with tandem stenosis and tried to explain how tandem stenosis formed at the carotid bifurcation. To our knowledge, it is the first study that addressed the tandem stenosis phenomenon by using a patient-specific carotid bifurcation data combined the *in vivo* imaging, *in vitro* histology, and *in silico* simulation together.

Nevertheless, there are still some limitations in this study to be discussed here. First of all, from the scope of the tandem stenosis, the stenosis occurs at carotid bifurcation ICA and CCA is not the only scenario of tandem stenosis, such as the previous mentioned ICA/MCA (El-Mitwalli et al., 2002; Kim et al., 2005; Rubiera et al., 2006) which is also a tandem stenosis related to ischemic stroke. Another limitation was due to the low frequency of tandem stenosis only one patient was investigated. The robustness of conclusion from this study requires further studies on more patients.

From methodology aspect, the healthy model was assumed by simply removing the stenosis part, the carotid shape remodeling during the plaque formation or CEA surgery had to be neglected due to lacking patient follow-up data, which would be useful to track the formation and treatment of tandem stenosis. In the operation of CEA, the tissue sample was cut open longitudinally during CEA. Therefore, the shape of histology image was out of its original shape, which might cause an error when register the histology with MR imaging. The resolution of MRI was another limitation of this study, as multi-sequence MR imaging data were acquired, it was a long-time scanning period, but the resolution of each sequence was relatively low. The resolution could significantly affect the precise of segmentation and 3D reconstructed model. In the CFD simulation, the elasticity of artery wall was ignored, the undeformed arterial wall would have a significant influence on the WSS result (Wang et al., 2019). The boundary conditions we used were a couple of massflow rate inlet and pressure outlet, the massflow rate inlet profile was extracted from this patient's 2D cine PC MR imaging. However, due to lack of patient-specific pressure data, and in order to study the flow distributed to ICA and ECA at different stenosis conditions, we had to assume both the ICA and ECA constant pressure outlet without specified flow distribution prescribed.

## Conclusion

*In vivo* MR imaging, *in vitro* histology, and *in silico* simulation were combined together in this patient case study. At the carotid bifurcation, the numerical evidence suggests that the stenosis at upstream CCA location was more likely to affect the downstream ICA location and lead to the formation of tandem stenosis. Tandem stenosis is proven as more vulnerable compared to a single-stenosis blood vessel, as the flow behavior became more complex and had lower WSS and turbulence areas after flowing through the tandem stenosis, which might cause the formation of new atherosclerosis or merge together forming a larger plaque. Therefore, we suggest, when planning CEA, the tandem stenosis should be considered to avoid secondary recurrent stenosis.

## Data Availability Statement

The datasets generated for this study are available on request to the corresponding author.

## Ethics Statement

This study was approved by the Metro South Human Research Ethics Committee (HREC/17/QPAH/181).

## Author Contributions

JW, PP, JM, YG, OR, and ZL contributed to the conception, design of the study, result discussion, and manuscript draft. TM and TL organized the data and sample collection. PP conducted the staining histology. JM performed the MR imaging processing and 3D modeling. JW carried out the computational simulation, data post-processing, and analysis. All authors have approved the final version of the manuscript.

### Conflict of Interest

The authors declare that the research was conducted in the absence of any commercial or financial relationships that could be construed as a potential conflict of interest.
